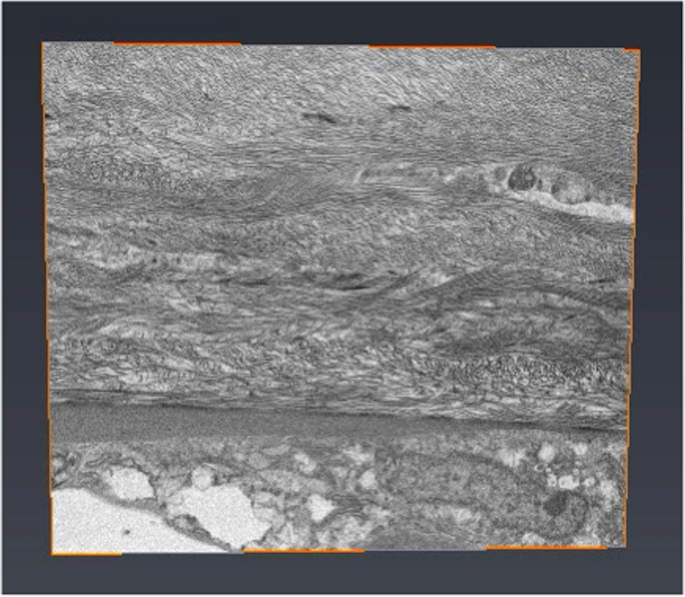# Developmental abnormalities in the cornea of a mouse model for Marfan syndrome

**DOI:** 10.1016/j.exer.2020.108001

**Published:** 2020-05

**Authors:** Eleanor M. Feneck, Rodrigo B. Souza, Philip N. Lewis, Sally Hayes, Lygia V. Pereira, Keith M. Meek

**Affiliations:** a Structural Biophysics Research Group, School of Optometry and Vision Sciences, Cardiff University, Maindy Road, Cardiff, CF24 4HQ, UK; b Department of Genetics and Evolutionary Biology, University of Sᾶo Paulo, Rua do Matᾶo, Sᾶo Paulo, Brazil

**Keywords:** Elastic fibres, Marfan syndrome, Fibrillin-1, Cornea, Mouse development, Extracellular matrix, AFS2, Leica automatic freeze substitution system, BSA, Bovine serum album, DN, dominant-negative, E, Embryonic day, *Fbn1*^*+/−*^, mgΔ^loxPneo^ mouse model, FD, Collagen fibril diameter, IFS, Collagen interfibrillar spacing, MFS, Marfan syndrome, OCT, Optical coherence tomography, PBS, Phosphate buffered saline, PFA, paraformaldehyde, SBF-SEM, Serial block face scanning electron microscopy, SAXS, Small-angle X-ray scattering, TEM, transmission electron microscopy, TGF-β, transforming growth factor-β, WT, Wild type

## Abstract

Elastic fibres provide tissues with elasticity and flexibility. In the healthy human cornea, elastic fibres are limited to the posterior region of the peripheral stroma, but their specific functional role remains elusive.

Here, we examine the physical and structural characteristics of the cornea during development in the mgΔ^loxPneo^ dominant-negative mouse model for Marfan syndrome, in which the physiological extracellular matrix of its elastic-fibre rich tissues is disrupted by the presence of a dysfunctional fibrillin-1 glycoprotein. Optical coherence tomography demonstrated a reduced corneal thickness in the mutant compared to wild type mice from embryonic day 16.5 until adulthood. X-ray scattering and electron microscopy revealed a disruption to both the elastic fibre and collagen fibril ultrastructure in the knockout mice, as well as abnormally low levels of the proteoglycan decorin. It is suggested that these alterations might be a result of increased transforming growth factor beta signalling. To conclude, this study has demonstrated corneal structure and ultrastructure to be altered when fibrillin-1 is disrupted and has provided insights into the role of fibrillin-1 in developing a functional cornea.

## Highlights

• mgΔ^loxPneo^ mice showed abnormalities in corneal thickness from embryonic development through to adulthood.
• Elastic fibres were evident from E16.5 in both the WT and mgΔ^loxPneo^ mouse corneas.
• Adult mgΔ^loxPneo^ mouse corneas exhibited a disorganised elastic fibre network with unusually high levels of branching.
• The disrupted collagen arrangement seen in adult mgΔ^loxPneo^ mice corneas is likely linked to lower levels of decorin in these corneas.

## 1 Introduction

Marfan syndrome is an autosomal dominant connective tissue disease caused by mutations in the *Fbn-1* gene that encodes the glycoprotein fibrillin-1, the major structural component of microfibrils. These fibres form a scaffold for elastin deposition during the formation of elastic fibres. Thus, the resultant mutation disrupts elastic fibre assembly and leads to a disorganisation of the extracellular matrix in tissues that are abundant in microfibrils, such as the cardiovascular, skeletal and ocular systems. Focussing on the optical system alone, Marfan syndrome is associated with lens dislocation (ectopia lentis), myopia (Gehle et al., 2017) and the presence of a thinned and flattened cornea (Konradsen and Zetterstrom, 2013; Maumenee, 1981; Sultan et al., 2002).

A precisely organised extracellular matrix is required for the cornea to be strong, transparent and precisely curved, and to enable it to successfully perform its primary functions of protecting the inner contents of the eye and transmitting and focussing incoming light for optimal vision. As has been demonstrated in numerous studies, disruption to the organisation of the collagen and proteoglycans within the extracellular matrix leads to alterations in the strength (Chakravarti et al., 1998), shape (Quantock et al., 2003) and transparency (Quantock et al., 2001) of the tissue. However, the functional importance of elastic fibres in the cornea is less well understood. Our recent studies have demonstrated species differences in the distribution of corneal elastic fibres, showing them to be localised to the posterior region of the peripheral stroma in the foetal and adult human cornea but existing as an extensive network of fibrillin-rich microfibril bundles throughout the mouse corneal stroma (Feneck et al., 2018; Lewis et al., 2016; Mohammed et al., 2018). Further knowledge has been gained from examination of corneal structure in the fibrillin-1 mgΔ^loxPneo^ mouse model for Marfan syndrome, in which the mutant FBN1 allele creates a truncated fibrillin-1 monomer. As the truncated fibrillin-1 disrupt microfibril formation and structure, there is a surge in TGF-β signalling that leads to pathological changes and the development of some of the phenotypic characteristics associated with the disease (Habashi et al., 2006; Neptune et al., 2003; Ramirez and Rifkin, 2009). In this model, the corneas of the adults were found to be thinner and flatter than those of age-matched wild type mice and to exhibit structural abnormalities in the organisation and distribution of their constituent collagen and elastic fibres (Lima et al., 2010; White et al., 2017). Thus, the study successfully demonstrated the important, and likely multifunctional role of elastic fibres in the adult mouse cornea.

In this study, we use the mgΔ^loxPneo^ mouse model containing an in-frame deletion of exons 19–24 in the FBN1 gene which leads to a truncated form of fibrillin-1 that exerts a dominant negative effect on microfibril formation (Lima et al., 2010). Heterozygous mice showed classical Marfan syndrome phenotypes, including aortic aneurysm and dissection, and hyperkyphosis. A variety of state-of-the-art microscopy and x-ray scattering techniques were used to characterise the physical appearance and structural characteristics of wild-type and mgΔ^loxPneo^ mice corneas, from the embryonic stage through to adulthood, in order to elucidate the role of elastic fibres in corneal development.

## 2 Methods

All tissue was obtained from the Department of Genetics and Evolutionary Biology, University of São Paulo (Brazil), in accordance with the ARVO Statement for the Use of Animals in Ophthalmic and Vision Research. All animal procedures and ethical regulations were approved by the Institutional Animal Care and Use Committee of the Instituto de Biociencias at USP. Protocol ID: CEA/IBUSP 272/2016 Process 16.1.632.41.7. Wild type (WT) female mice were crossed with heterozygous Marfan syndrome male mice from the same genetic background. Genotyping of the litter was carried out as described previously to identify mgΔ^loxPneo^ mouse model (herein referred to as *Fbn1*^*+/−*^) and WT mice (Lima et al., 2010). The animals were sacrificed with exsanguination under general anaesthesia (0.01/100 mg Ketamine® and Xylazine® (4:1). Pairs of whole mouse eyes were enucleated and immediately transferred and stored in 0.5% paraformaldehyde (PFA) and transported to the UK for structural analysis. One-hundred and twenty eyes were obtained from *Fbn1*^*+/−*^ mice and wild type equivalents (aged E12.5, E14.5, E16.5, E18.5 and 6-month adults, with n = 24 at each age).

### 2.1 Optical Coherence Tomography (OCT)

Using the same methodology as described previously (White et al., 2017), a near-infrared (NIR) bespoke OCT microscope was used to determine corneal thickness and corneal curvature in 60 un-paired eyes from *Fbn1*^*+/−*^ mice at the following stages of development: E12.5, E14.5, E16.5, E18.5 and 6-months (adulthood). Corresponding data was obtained from WT eyes, with the exception of developmental stage E18.5, which was limited to examination of 3 un-paired samples.

The bespoke OCT microscope set-up comprised a light source with a central wavelength of 1040 nm and a bandwidth of 70 nm (1-M-ASE-HPE-S; NP Photonics, Tucson, AZ, USA) which was coupled through a 2 x 2 optical fiber coupler (FC; FOBC-2-64 ± 100-20-L-H64f-2; AFW Technologies, Hallam, Victoria, Australia) and connected to an imaging head and reference arm. The imaging head contained an achromatic off-axis parabolic reflector to collimate the fiber output beam to 2 mm diameter (RC02APC-P01; Thorlabs, Ely, UK) with 2-D (XY) optical scanners (6210HBM60/6102103R; Cambridge Technology Division, GSI Group GmbH, Muenchner, Germany) and a broad-band near-infrared telecentric scan lens (LSM02BB; Thorlabs). The reference arm contained a polarization controller (PC; FPC560, Thorlabs), a reflecting collimator (RC08APC-01; Thorlabs), an adjustable aperture, a precision near-infrared retroreflector (RR; 1 Arcsec Gold, Edmund Optics, York, UK) and a glass compensation block (CB; LSM02DC; Thorlabs) to correct for dispersion in the scan lens. Reflected light from the specimen and reference arm was combined in a spectrometer. The camera within the OCT microscope was a 47 kHz Goodrich SU-LDH-1.7 (UTC Aerospace Systems, Arlington, VA, USA). The OCT was controlled by LabView (National Instruments, Newbury, UK) software and spectra.

Prior to data collection each eye was placed onto a glass slide and secured by Blu Tack® to ensure that the sample remained stable and in the correct position for scanning, with the corneal surface facing upwards and its centre aligned with the position of the scan lens. Corneas were hydrated with 0.5% PFA to avoid drying and surface wrinkling. Datasets of 1000 images were collected at a 1024 x 1024 pixel resolution at different scanning angles (5.61°, 11.2° and 16.8°) and processed into a 3-D volume imaged by spectral resampling and Fourier transformation. Datasets were then processed and analysed using MATLAB (Mathworks, Cambridge, UK) and ImageJ/Fiji software (Wayne Rasband, U.S. National Institutes of Health, Maryland, USA) to produce measurements of corneal curvature and thickness using the same method described previously (White et al., 2017).

### 2.2 Small angle X-Ray scattering (SAXS)

Small-angle x-ray scattering patterns were obtained at the I22 beamline (Diamond Light Source, Oxfordshire, UK) from 6 un-paired WT and 6 un-paired Fbn1^+/−^ samples at each of two developmental stages, E18.5 and 6-month adult. Immediately before data collection, the cornea (with an approximately 1 mm scleral rim) was manually dissected from each eye. The corneo-scleral samples were then wrapped tightly in Clingfilm™ to prevent tissue dehydration, and secured within a Perspex sample holder with Mylar windows. The sample holder was positioned on the beamline such that the anterior surface of the central cornea was perpendicular to the direction of the incident x-ray beam. A series of 0.1 s exposures to a 0.1 nm wavelength x-ray beam, focussed to measure 200 × 200 μm at the cornea, were used to generate x-ray scatter patterns at 0.3 mm intervals across a 7.2 mm × 7.2 mm region that encompassed the cornea and surrounding sclera. The resulting x-ray patterns were recorded on a Pilatus P3-2M detector.

Analysis of the scatter patterns was undertaken using SAXS4COLL software (Abass et al., 2017) to generate measurements of fibril diameter and collagen interfibrillar spacing. The measurements of interfibrillar spacing represent the centre-to-centre spacing of collagen fibrils assuming a liquid-like packing arrangement (Worthington and Inouye, 1985).

### 2.3 Electron microscopy

Elastic fibres were stained with tannic acid and uranyl acetate (Simmons and Avery, 1980). Three un-paired WT and 3 un-paired *Fbn1*^*+/−*^ corneas were collected from every age between E12.5 and adult, dissected into quadrants and fixed in Karnovsky's fixative (2.5% glutaraldehyde, 2% paraformaldehyde, 0.1 M cacodylate buffer pH 7.2). Karnovsky's-fixed quadrants were washed in sodium cacodylate buffer 3 times over 10 min and in distilled water (dH₂0) for 5 min. Samples were post-fixed in 1% osmium tetroxide for 1 h, washed with dH₂0 three times over 20 min before being transferred to 0.5% filtered tannic acid (TA) in dH₂0 for 2 h. Samples were washed with dH₂0 three times over 30 min and left overnight in 2% aqueous uranyl acetate (UA). Samples were then dehydrated in a 70–100% ethanol series. Samples were further en bloc stained with 1% UA for 2 h, followed by lead acetate in 1:1 ethanol and acetone for 2 h. The samples were washed with 1:1 ethanol acetone twice over 20 min and then washed three times over 20 min with 100% acetone. Samples were infiltrated with 1:1 acetone and Araldite resin (Araldite monomer CY212 and DDSA hardener) for 1 h. BDMA accelerator was added to the pre-made Araldite resin, making continuous resin changes to the samples every 2 h until 6 changes had been made. The samples were embedded and polymerised at 60 °C for 48 h.

### 2.4 Serial block face-scanning electron microscopy (SBF-SEM)

Three samples for each age were mounted onto a Gatan specimen pin and coated with a silver conductive epoxy adhesive (TAAB laboratories). Each pin was sputtered with gold to a thickness of 8 nm using a Leica ACE 200 gold coating unit and placed inside the Zeiss Sigma VP FEG SEM equipped with a Gatan 3View system. The block face surface was imaged on the SEM at 3.5 kV at a pixel resolution of 4 nm and a dwell time of 8 μs and approximately 1000 image slices were acquired from the specimen block every 50 nm by automated serial sectioning. The data sets were recorded at 4096 x 4096 (4K) in Gatan format dm4 files then batch converted to TIFF format.3-D reconstructions of the datasets using the isosurface or segmentation functions were composed using Amira 6.4 software (FEI, Mérignac, France).

### 2.5 Transmission Electron Microscopy (TEM)

Ultrathin sections were cut (90 nm) from all of the SEM 3View blocks using the Leica UC6 ultra-microtome, collected on 300 hexagonal copper grids and imaged using the JEOL 1010 transmission electron microscope at an accelerating voltage of 80 KV, fitted with a Gatan Orius 1000 TEM camera (Gatan, Abingdon, England).

### 2.6 Immuno-electron microscopy

To investigate the mechanisms of collagen fibril disorganisation, immunogold electron microscopy labelling of decorin was undertaken in the central region of 6 adult WT and 6 adult Fbn1^+/−^ corneas. Samples stored in 0.5% paraformaldehyde were further stored in 0.2M Sörensen phosphate buffer (pH 7.4). Samples were transferred into 30% ethanol overnight before being placed into the Leica AFS2 automatic freeze substitution system (AFS2) (Leica Microsystems, Wetzlar, Germany), to gradually lower the temperature of the samples. Once the temperature had reached −15 °C, samples were immersed in 55% ethanol for 1 h. The temperature was further reduced to −30 °C with 75% ethanol for 1 h, then −35 °C with 100% ethanol for 1 h twice. The ethanol was replaced with 30% HM20 for 1 h, gradually increasing the concentration of HM20 every hour (60% and 100%). Samples were left in 100% HM20 overnight, placed in a fresh 100% HM20 solution three times before being UV light polymerised for 60 h.

Ultrathin sections (90 nm) were cut using a Leica UC6 ultramicrotome and collected on hexagonal gold 300 grids. Grid samples were rehydrated with an incubation solution containing Tris-acetate buffer (pH 7.2) in 1% bovine serum album (BSA), 1% protease inhibitor and Chondroitinase ABC for 1 h. The samples were washed ten times in Tris-acetate buffer for 10 min, and further incubated with phosphate buffered saline (PBS) for 10 min. They were placed on 50 mM ammonium chloride in PBS (pH 7.4) for 5 min, washed 10 times for 10 min with PBS and then placed in 1% BSA in PBS twice over 10 min. Grids were incubated with 1:10 mouse monoclonal decorin primary antibody (gifted by Professor Claire Hughes, Cardiff University, School of Biosciences) diluted in blocking buffer for 2 h at room temperature. They were washed 6 times for 2 min with blocking buffer and further incubated with the goat anti-mouse 10 nm gold-conjugated secondary antibody (Abcam, Cambridge, UK) at 1:20 concentration diluted in blocking buffer for 1 h at room temperature. Samples were further washed twice for 2 min in blocking buffer, four times in PBS and 10 times with double distilled water. Finally, the sections were counterstained with uranyl acetate and lead and analysed on a JEOL 1010 transmission electron microscope at an accelerating voltage of 80 KV. The microscope was fitted with a Gatan Orius 1000 TEM camera (Gatan, Abingdon, England).

### 2.7 Statistical analysis

All statistical analyses were performed in MATLAB R2018a. A Kolmogorov-Smirnov test was used to test normality. If the data had a normal distribution, a two-sample *t*-test was carried out. If data was skewed, a Mann-Whitney *U* test was conducted. Any p-value of less than 0.05 was determined to be statically significant.

## 3 Results

### 3.1 OCT

An absence of distinguishable corneal boundaries by OCT at E12.5 prevented accurate measurement of corneal thickness and radius of curvature. At E14.5, measurements of corneal thickness revealed no significant difference between the Fbn1^+/−^ and WT mice. However, from E16.5 onwards, the central corneal thickness of the Fbn1^+/−^ mice was significantly lower than that of age-matched WT mice (Fig. 1 and Table 1). Accurate radius of curvature measurements could only be obtained from the adult mice. The results showed corneal radius of curvature to be on average higher in the Fbn1^+/−^ mice (1.26 ± 0.20 mm) than the WT mice (1.19 ± 0.32 mm). However, the limited sample size and relatively large within-group variation in this particular parameter resulted in an insufficient study power to warrant meaningful statistical analysis.

**Fig. 1:**
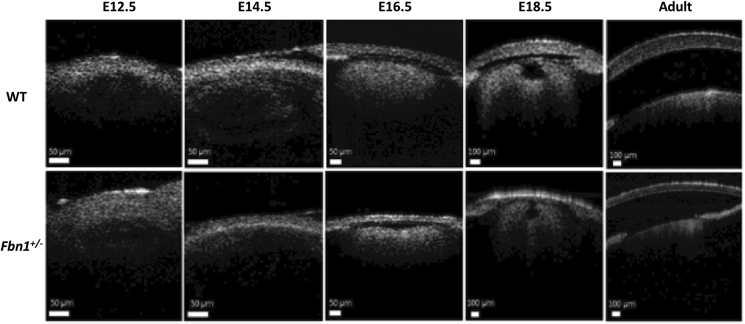
**Optical Coherence Tomography (OCT) images of wild type (WT) and the fibrillin-1 (Fbn1**^**+/−**^**) mouse corneas at various stages of development.** OCT analysis was used to calculate corneal thickness and radius of curvature. From embryonic (E) day 16.5 onwards, the central corneal thickness of the *Fbn1*^*+/−*^ mice was lower than that of the age-matched WT mice. Adult *Fbn1*^*+/−*^ mice typically exhibited thinner and flatter corneas than their WT counterparts.

**Table 1:** Mean central corneal thickness in embryonic and adult wild type (WT) and fibrillin-1 knockout (Fbn1^+/−^) mouse corneas.

Age	Average Central Corneal Thickness WT (μm)	Average Central Corneal Thickness FBN1bn^+/−^ (μm)	Statistical Significance (p-value)	Number of Fbn1^+/−^ mice analysed	Number of WT mice analysed
Adult	197.17±9.5	148.29±7.3	0.0022	7	7
E18.5	124.05±3.3	83.28±11.1	0.0238	6	3
E16.5	42.68±8.3	26.80±3.6	0.0003	7	6
E14.5	25.4±4.6	23.1±1.6	0.1564	6	6
E12.5	N/A	N/A	N/A	6	6

### 3.2 Small angle X-ray scattering (SAXS)

**Fig. 2:**
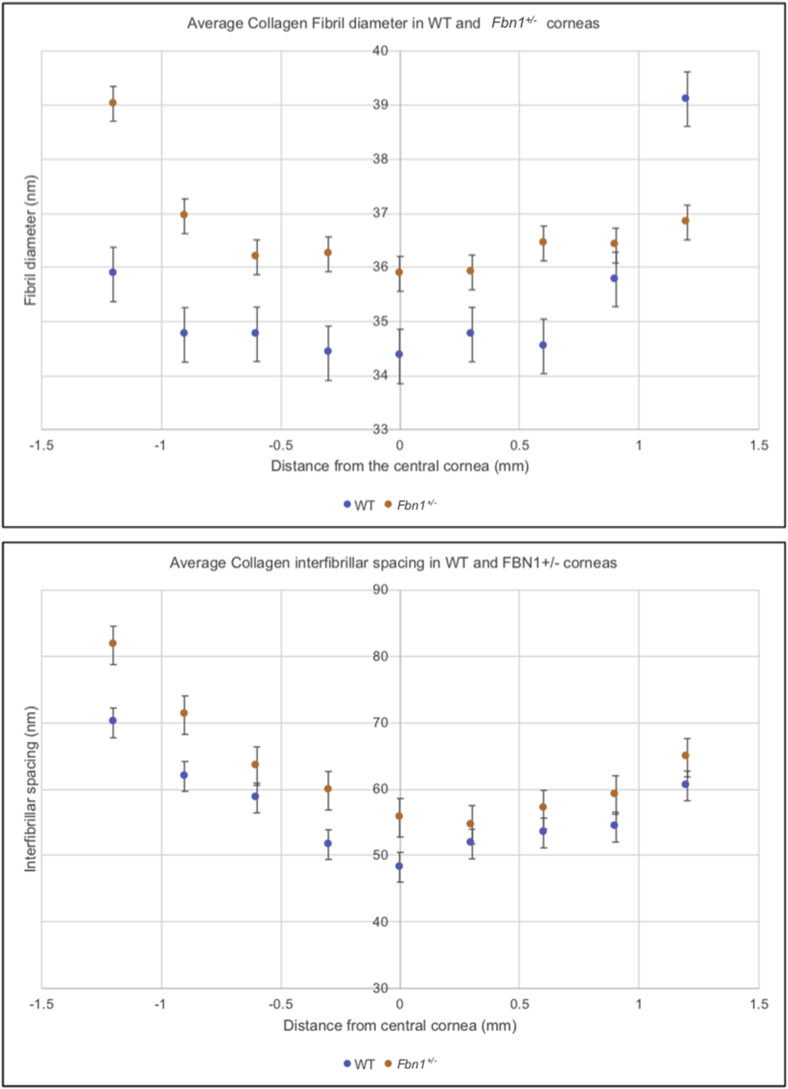
**Small angle x-ray scattering (SAXS) measurements from adult wild type (WT) and a fibrillin-1 knockout (Fbn1**^**+/−**^**) mouse cornea.** Average collagen interfibrillar spacing and collagen fibril diameter increased from the central cornea to the peripheral cornea in both the adult WT and Fbn1^+/−^ mouse models. Bars represent standard error.

Only the adult corneal tissue could be analysed with SAXS as the embryonic corneas did not produce a SAXS pattern for data collection. In both WT and Fbn1^+/−^ adult corneas there was an increase in collagen interfibrillar spacing (IFS) and collagen fibril diameters (FD) from the central to the peripheral cornea, producing a U-shaped distribution on the radial plots (Fig. 2). The average central IFS was significantly greater in the Fbn1^+/−^ mouse corneas (55.7 nm) than in the WT tissue (48.3 nm) (p = 0.04). However, central collagen FD was not significantly different between the WT and Fbn1^+/−^ mouse cornea (p = 0.07). These results demonstrated a change in collagen ultrastructure in the adult Fbn1^+/−^ corneas compared to the adult WT, with significant changes in central collagen IFS.

### 3.3 Elastic fibre imaging

**Fig. 3:**
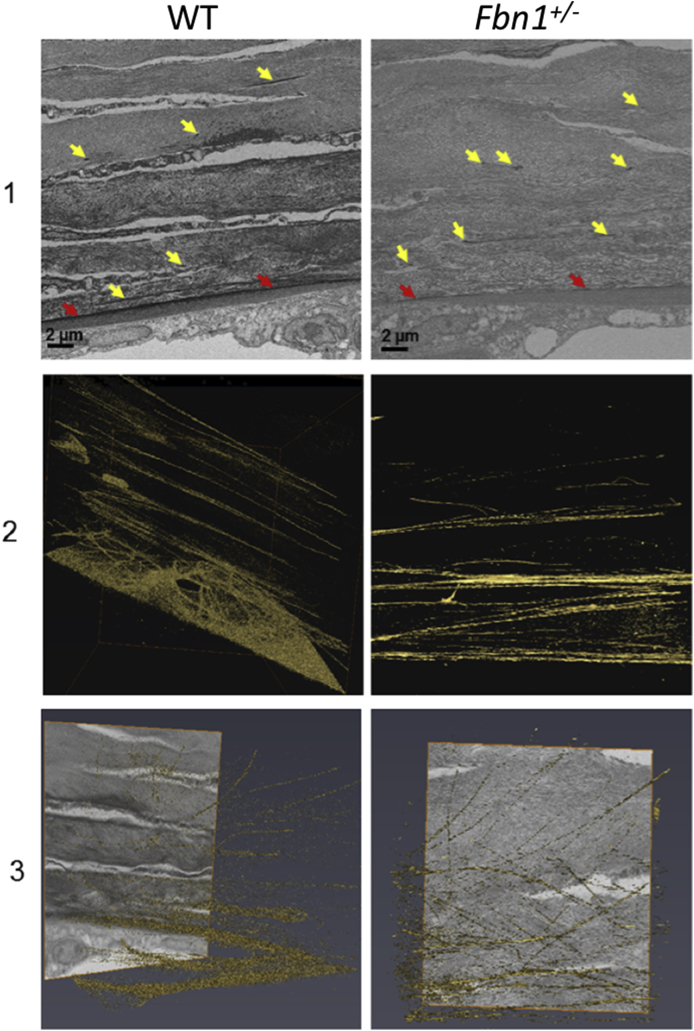
**Serial block face-scanning electron microscopy (SBF-SEM) of the adult wild type (WT) and fibrillin-1 knockout (Fbn1**^**+/−**^**) mouse posterior corneal stroma.** The posterior corneal stroma was analysed with SBF-SEM. Elastic fibres were enhanced anterior to Descemet's membrane (red arrows), with individual elastic fibres throughout the corneal stroma (yellow arrows) (panel 1). Elastic fibres were rendered with an automated isosurface function in Amira (gold) (panels 2–3). Elastic fibres had a parallel arrangement within the WT cornea (panel 2–3). This organisation was reduced, with elastic fibres that appeared more disorganised within the Fbn1^+/−^ mouse model (panel 2–3) compared to the WT. For greater details of the 3-D models refer to supplementary videos 1 and 2 (Fig. S1).

**Fig. 4:**
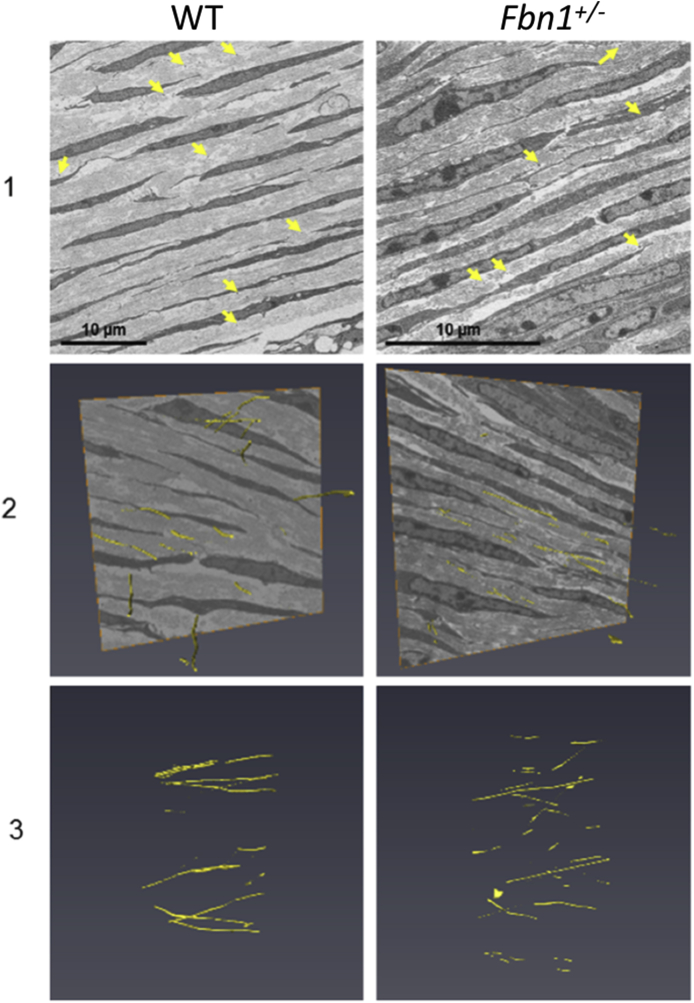
**Serial block face-scanning electron microscopy (SBF-SEM) of the E18.5 wild type (WT) and fibrillin-1 knockout (Fbn1**^**+/−**^**) mouse posterior corneal stroma.** The posterior stroma was chosen for analysis with SBF-SEM. Elastic fibres (yellow arrows) were detected in cross-section between keratocytes within the extracellular matrix (panel 1). Elastic fibres were rendered with a manual segmentation tool (panel 2–3). Elastic fibres were present as individual structures. It could not conclusively be determined if there were differences in elastic fibre structure and distribution in the WT and Fbn1^+/−^ mouse model at E18.5 of embryonic development (panel 2–3).

**Fig. 5:**
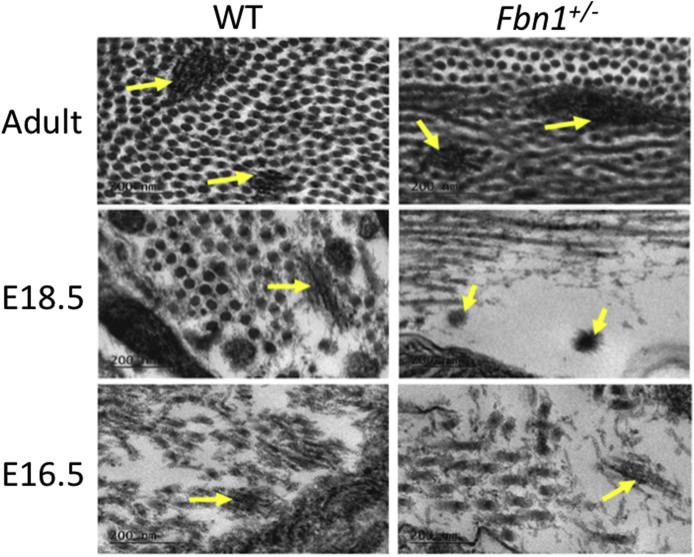
**Transmission electron microscopy (TEM) imaging of the wild type (WT) and fibrillin-1 knockout (Fbn1**^**+/−**^**) elastic fibres in the posterior corneal stroma.** Elastic fibres were imaged at high resolution with TEM. Elastic fibres were imaged as fibrous structures with an enhanced contrast within the posterior corneal stroma extracellular matrix of both WT and Fbn1^+/−^ corneas (yellow arrows). Elastic fibres were only identified from E16.5 of corneal development, no elastic fibres were identified in any younger corneal tissue. The fibres were composed of bundles of smaller microfibrils ~10–12 nm in diameter, having a similar morphology to fibrillin-microfibrils. Scale bars = 200 nm.

**Fig. 6:**
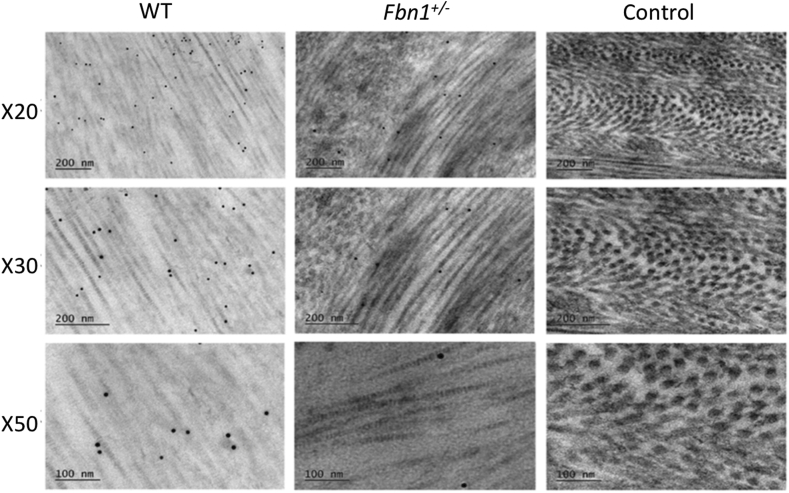
**Transmission Electron Microscopy (TEM) imaging of the wild type (WT) and fibrillin-1 knockout (Fbn1**^**+/−**^**) corneas with decorin immunogold particle labelling.** Decorin positively labelled in the WT and Fbn1^+/−^ corneal stroma, with no positive staining detected in the control sections that contained no primary antibodies. Decorin labelled between and on top of collagen fibrils in both models. Quantitative analysis of the gold particles was undertaken to determine any differences in staining between the WT and the Fbn1^+/−^ adult corneas.

In the adult WT cornea, microfibrils were enhanced directly anterior to Descemet's membrane with longitudinal fibres that presented within the posterior corneal stroma, branching into different fibres but generally remaining parallel to the corneal surface. A much less organised elastic fibre arrangement was seen in the adult Fbn1^+/−^ mouse cornea, with elastic fibres branching off in many different directions (in-plane and out-of-plane) within the posterior corneal stroma (Fig. 3 and Fig. S2). Unfortunately, due to the very limited elastic fibre content of the embryonic tissue and the narrowness of the fibres, the automated isosurface function could not be used for these images. Instead, a manual reconstruction tool was used to trace the elastic fibres in the developing cornea. Elastic fibres were identified within the posterior corneal stroma of both the E18.5 WT and Fbn1^+/−^ mouse corneas, with no obvious structural differences between the two (Fig. 4). TEM analysis identified elastic fibres in high resolution from E16.5 of corneal development (Fig. 5). The elastic fibres were composed of bundles of microfibrils ~10–12 nm in diameter, with a morphology similar to fibrillin-rich microfibrils (Fig. 6).

### 3.4 Immunogold-electron microscopy analysis of decorin

Decorin was positively labelled in both the WT and Fbn1^+/−^ central mouse corneas, with no gold labelling present in the control sections that contained no primary antibody (Fig. 6). Gold particle labelling of the proteoglycan core of decorin was found to be closely associated with the surface of the collagen fibrils. Quantitative analysis of the gold particle labelling found the Fbn1^+/−^ mouse corneas to have significantly less decorin gold labelling compared with the WT corneas (p = 0.00008).

## 4 Discussion

Our previous studies of the adult mgΔ^loxPneo^ mouse model, which exhibits some of the phenotypic characteristics associated with Marfan syndrome, identified abnormalities in corneal geometry (thinner and flatter corneas) that were accompanied by irregularities in the organisation of both collagen and elastic fibres within the corneal stroma (White et al., 2017). These results raised questions about the stage at which these abnormalities occur and the potential role of elastic fibres in corneal development (Lewis et al., 2016). To gain further insight into this issue, a thorough investigation was conducted into the physical and structural properties of embryonic and adult wild type mice corneas and age-matched corneas from mgΔ^loxPneo^ mice.

Optical coherence tomography measurements of embryonic mouse corneal thickness and radius curvature were technically challenging, and in many cases not possible, due to the lack of clearly discernible corneal boundaries at this early stage of development. In the adult mice, the corneas of the Fbn1^+/−^ mice were typically flatter than those of WT mice but meaningful statistical analysis of radius of curvature measurements was prevented by the limited sample size and relatively large within-group variability. However, in accordance with our previous findings (White et al., 2017), we observed that adult Fbn1^+/−^ mice have a thinner cornea than WT controls. Further to this, our study revealed that the abnormalities in corneal thickness in the Fbn1^+/−^ mice are evident not only in adulthood but also during embryonic development (from E16.5 onwards). In an attempt to determine the cause of this thinning, x-ray scattering was used to examine the structure of the cornea in the embryonic and adult Fbn1^+/−^ and WT mice.

Unfortunately, measurements of collagen FD and IFS could not be obtained from the embryonic corneas as they failed to produce discernible x-ray scatter patterns. This most probably resulted from there being an insufficient amount of organised collagen fibrils within the developing corneas to produce a detectable x-ray scatter pattern. Previous studies analysing mouse postnatal development demonstrated only a weak scatter pattern from day 10 post-birth, indicating that older developmental ages are required to retrieve a scatter pattern (Sheppard et al., 2010). Similarly to the previous studies of the Fbn1^+/−^ mice, x-ray scattering measurements from adult Fbn1^+/−^ mice revealed that the diameter of the collagen fibrils did not differ from that of the WT corneas but the reduced corneal thickness was accompanied by the presence of more widely separated collagen fibrils. This suggests that overall there must be fewer collagen lamellae in the adult Fbn1^+/−^ mice compared to the same aged WT mice.

In addition to the abnormalities in collagen fibril spacing that were seen in the adult Fbn1^+/−^ mice, 3-D reconstructions of SBF-SEM datasets highlighted significant abnormalities in their elastic fibre network. In contrast to the adult WT mouse, where elastic fibres were concentrated directly anterior to Descemet's membrane with individual elastic fibres organised in a parallel arrangement to one another, the elastic fibres in adult Fbn1^+/−^ corneas appeared disorganised and branched in many directions. The appearance of elastic fibres running in many directions parallel to the surface of the cornea and often bifurcating is also described in previous findings of the mouse (Hanlon et al., 2015). It is not clear precisely at what stage the formation of the elastic fibre network in the Fbn1^+/−^ mouse cornea deviates from that of the WT mouse as, due to the complexity and time-consuming nature of the technique, it was not feasible to generate and analyse SBF-SEM data from each of the developmental stages. However, TEM revealed that in both the WT and Fbn1^+/−^ mice, the first appearance of elastic fibres occurred at E16.5 and the fibres continued to be observed at each of the examined embryonic stages through to adulthood. The 3-D reconstructions of the developing elastic fibres in the embryonic cornea at E18.5 did not show a significant difference in organisation between the WT and the Fbn1^+/−^ models, suggesting that fibrillin-1 does not play a role in the initial development of the cornea but does contribute to corneal structural development from E16.5 onwards. However, a disruption to the elastic fibre system is identified in the adult Fbn1^+/−^ mouse cornea. This could be explained by fibrillin-1 having a more prominent role in adult tissues for structural support, compared with fibrillin-2, which has a more significant role in embryonic tissues (Zhang et al., 1995). The localisation of fibrillin-1 in the prenatal mouse cornea is also increased in E16.5 corneas compared with E12.5 corneas, which further supports that fibrillin-1 has a more prominent role in corneal development from E16.5 onwards (Shi et al., 2013). To further understand if the cornea is affected by the development of elastic fibres, a knockout mouse model of fibrillin-2 could be explored to understand if the initial events of developing the cornea are altered by disruptions in early elastic fibre assembly (Zhang et al., 1995).

The Fbn1^+/−^ mouse model has a dysfunctional *Fbn-1* allele that generates a truncated fibrillin-1 glycoprotein that disrupts microfibril assembly/structure, and thus function. One of these functions is to bind to latent TGF-β binding protein (LTBP) to prevent TGF-β surges, which cause pathological alterations to the extracellular matrix. The disrupted microfibrils lead to a subsequent surge of active TGF-β, which causes changes to the extracellular matrix structure and function. Previous studies have also shown that an increase in TGF-β signalling in Marfan syndrome can suppress type I collagen production via the induction of the transcription factor CUX1 (Fragiadaki et al., 2011). This pathway could explain the reduction of collagen observed in the Fbn1^+/−^ mouse cornea. The reduction of collagen might also be explained by TGF-β surges increasing metalloproteinases 2 and 9, which are both known to degrade elastic fibres and collagen fibrils (Gomes et al., 2012). Furthermore, alterations to the primary structure of type I collagen, and resulting abnormalities in crosslink formation which have been described in some MFS patients (Byers et al., 1981), may render the tissue more susceptible to the action of such degradative enzymes. The results presented in this study demonstrate that disruption to fibrillin-1 alters collagen fibril organisation in the cornea. Alterations to the collagen fibril network have also been demonstrated in other tissues, such as the periodontal ligaments (Ganburged et al., 2010) and aorta (Ju et al., 2014) in two different fibrillin-1 knockout mouse models and in the aorta of patients with MFS (Lindeman et al., 2010). The mechanisms of how this collagen organisation is lost need to be elucidated to further interpret the role of the elastic fibres in the extracellular matrix.

Decorin is a member of the family of small leucine-rich proteoglycans (SLRPs) that plays a key regulatory role in collagen fibril organisation and fibrillogenesis within the cornea (Kahari et al., 1991; Li et al., 2006; Reed and Iozzo, 2002; Westergren-Thorsson et al., 1993) and is the major SLRP in the adult mouse cornea. The proteoglycan also has multiple non-structural functions in that it binds various growth factors including TGF-β and is also known to modulate a wide range of signalling pathways associated with matrix assembly (Chen et al., 2015). To determine if decorin levels were altered, immunogold electron microscopy particle labelling of the decorin core protein in both WT and Fbn1^+/−^ mouse corneas was carried out. Quantitative analysis demonstrated that there was a significant reduction of decorin gold labelling in the Fbn1^+/−^ images when compared to the WT images. Studies have shown that an enhancement in TGF-β levels occurs in Marfan syndrome, causing decorin levels to decrease through high and low affinity binding of the growth factor to the decorin core protein (Superti-Furga et al., 1992; Yamaguchi et al., 1990). The current Marfan mouse model is linked with the pathway associated with elevated levels of active TGF-β. This could suggest one possible explanation for the significant reduction of decorin labelling observed in the Fbn1^+/−^ stroma. However, deficiencies in mRNA synthesis of decorin and reduced expression have also previously been reported to occur in neonatal Marfan syndrome with abnormalities in the fibrillin gene (Superti-Furga et al., 1992). Thus, it could also be the case that the lack of decorin labelling is a reflection of reduced synthesis of the proteoglycan.

Clearly further work is required to fully determine levels of decorin in the Marfan model and its interaction with cytokines including TGF-β. Although the precise cellular mechanisms that contributed to the apparent low levels of decorin seen in the Marfan model remain unclear, the TEM observation showing the occasional enlarged abnormal fibre would seem to fit with subtle changes in collagen fibre morphology previously described in a decorin deficient mouse cornea by Zhang et al. (2009). In addition, other proteoglycan and collagen regulatory molecules should be analysed to further determine the exact mechanism that disrupts corneal stromal architecture in the Fbn1^+/*−*^ mouse model.

This research has provided a comprehensive structural study of corneal development in the Fbn1^+/−^ mouse model from E12.5 to E18.5, as well as the analysis of the adult system. The cornea has many structural changes post-birth, analysis of these post-natal ages could also reveal more information about the developing elastic fibre system in the cornea. Future experiments should focus on understanding the underlying mechanistic, biomechanical and functional changes that are present within the Fbn1^+/−^ mouse model to further understand the role of elastic fibres within the cornea. This study provides novel evidence of disruptions in corneal shape, thickness and ultrastructure from embryonic day 16.5 onwards in the Fbn1^+/−^ mouse model for Marfan syndrome and our results suggest that the elastic fibre network has a functional role in the successful development of the corneal stroma.

## Financial disclosure

No Author has a financial or proprietary interest in any material or method enclosed.

## Declaration of competing interest

The Authors have no conflicts of interest to disclose.

## Figures and Tables

**figs1:**
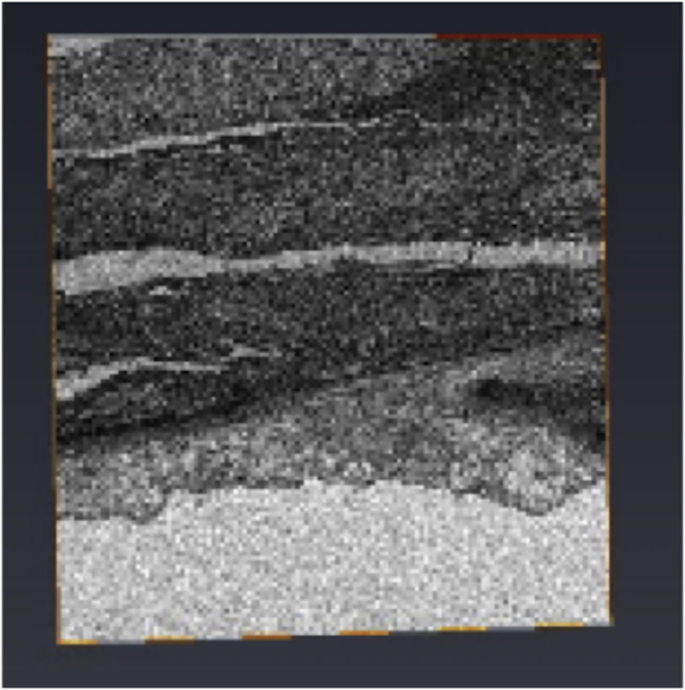


**figs2:**